# Supplementary material for: Identification of serum protein biomarkers in individuals with Niemann-Pick disease, type C1
Source: medRxiv. 2026 Jan 18:2026.01.12.26343721. Preprint. [Version 1] doi: 10.64898/2026.01.12.26343721 (PMC12930457; doi:10.64898/2026.01.12.26343721)
Supplement: Supplement 2 [file media-2.pptx]

## Slide 1
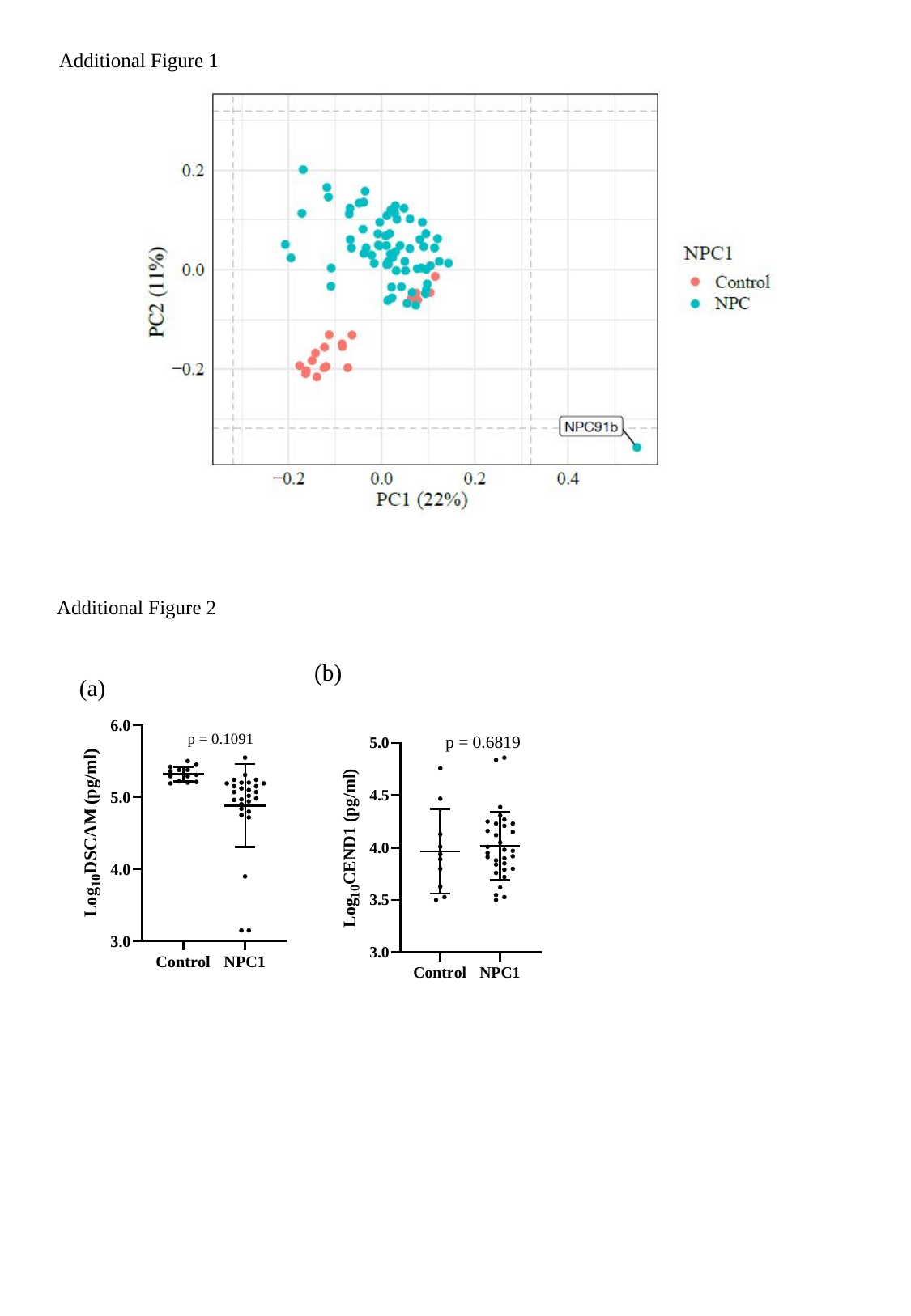

Additional Figure 1
Additional Figure 2
(b)
(a)
p = 0.1091

## Slide 2
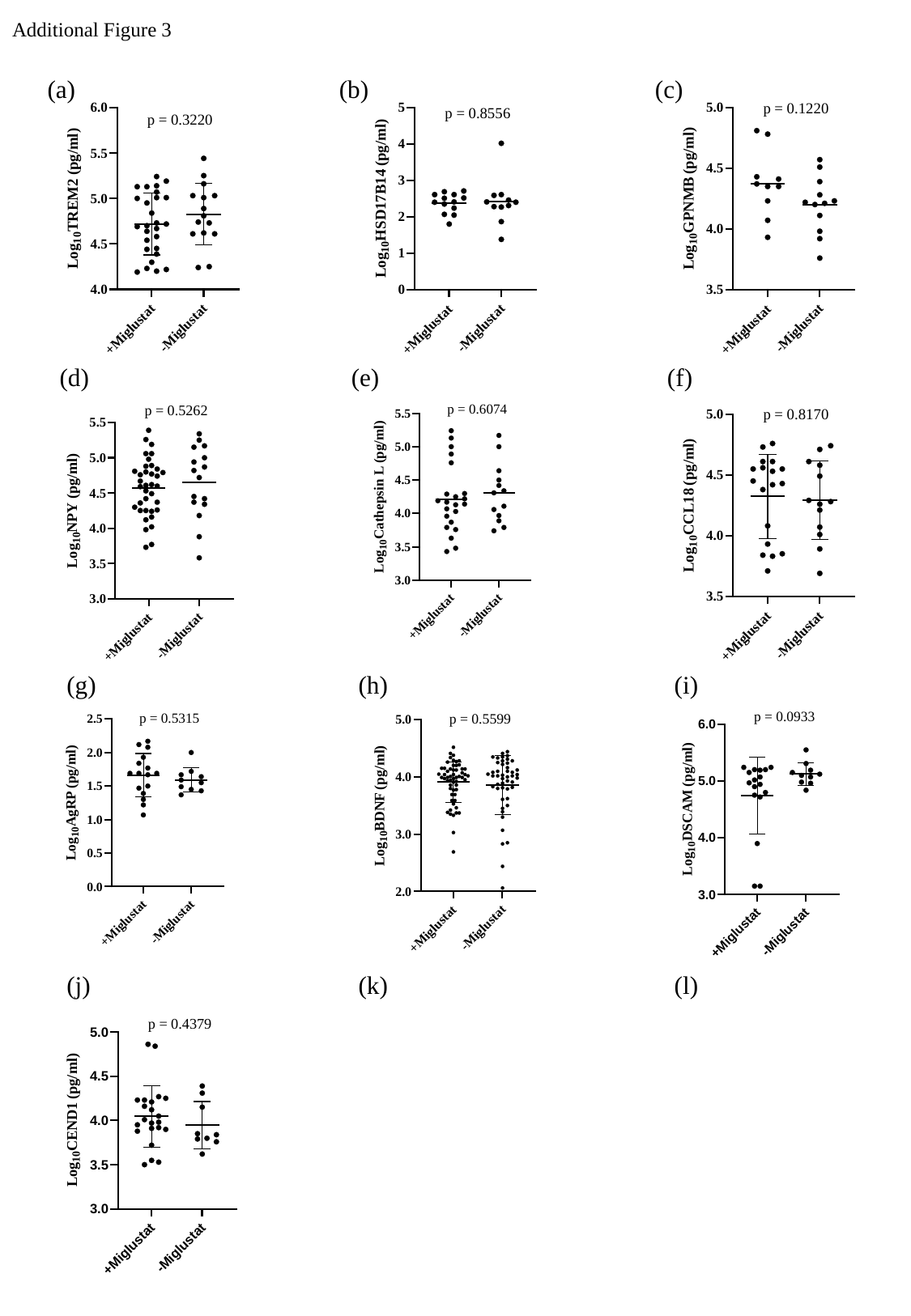

Additional Figure 3

## Slide 3
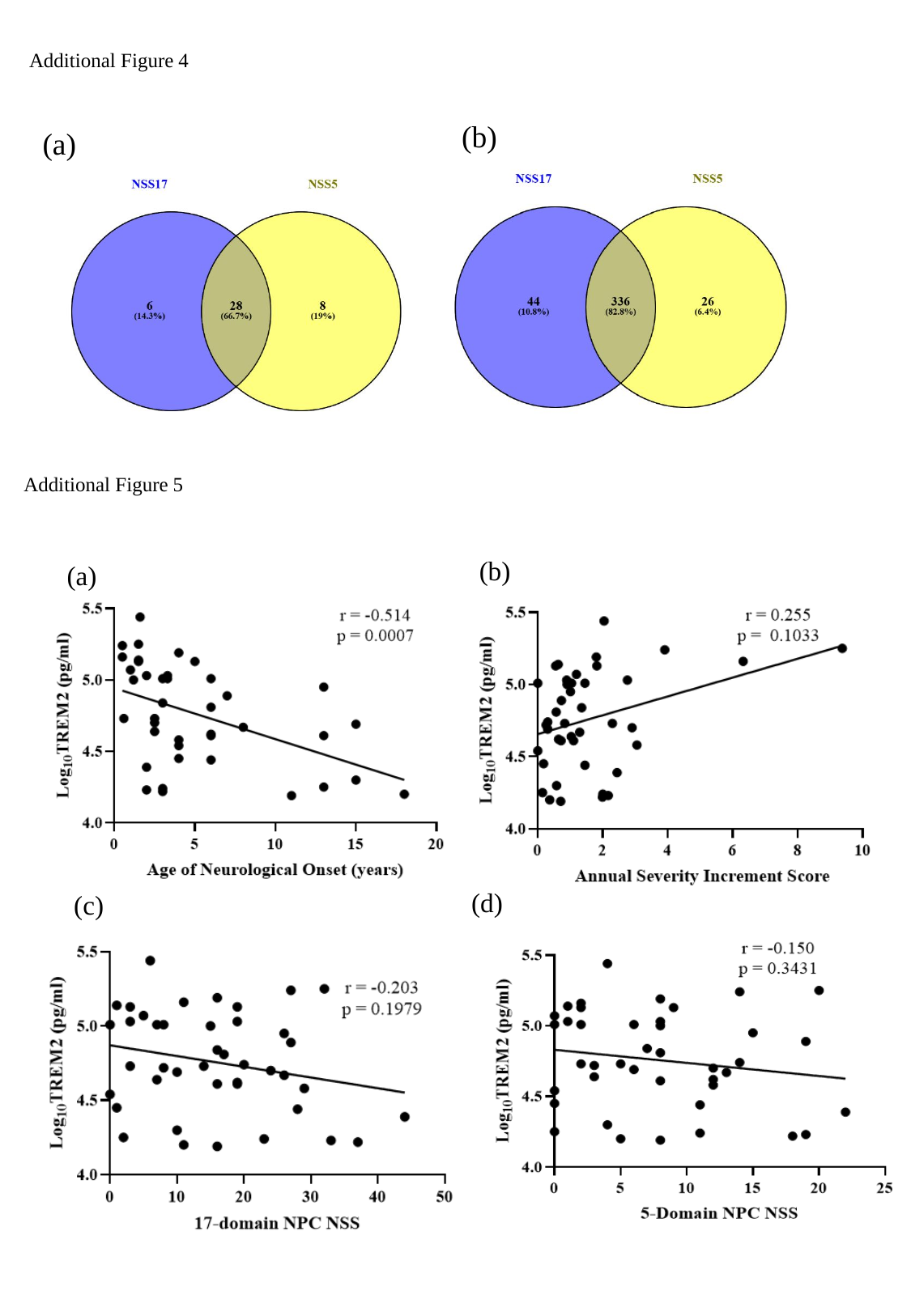

Additional Figure 4
(b)
(a)
Additional Figure 5
(b)
(a)
(d)
(c)

## Slide 4
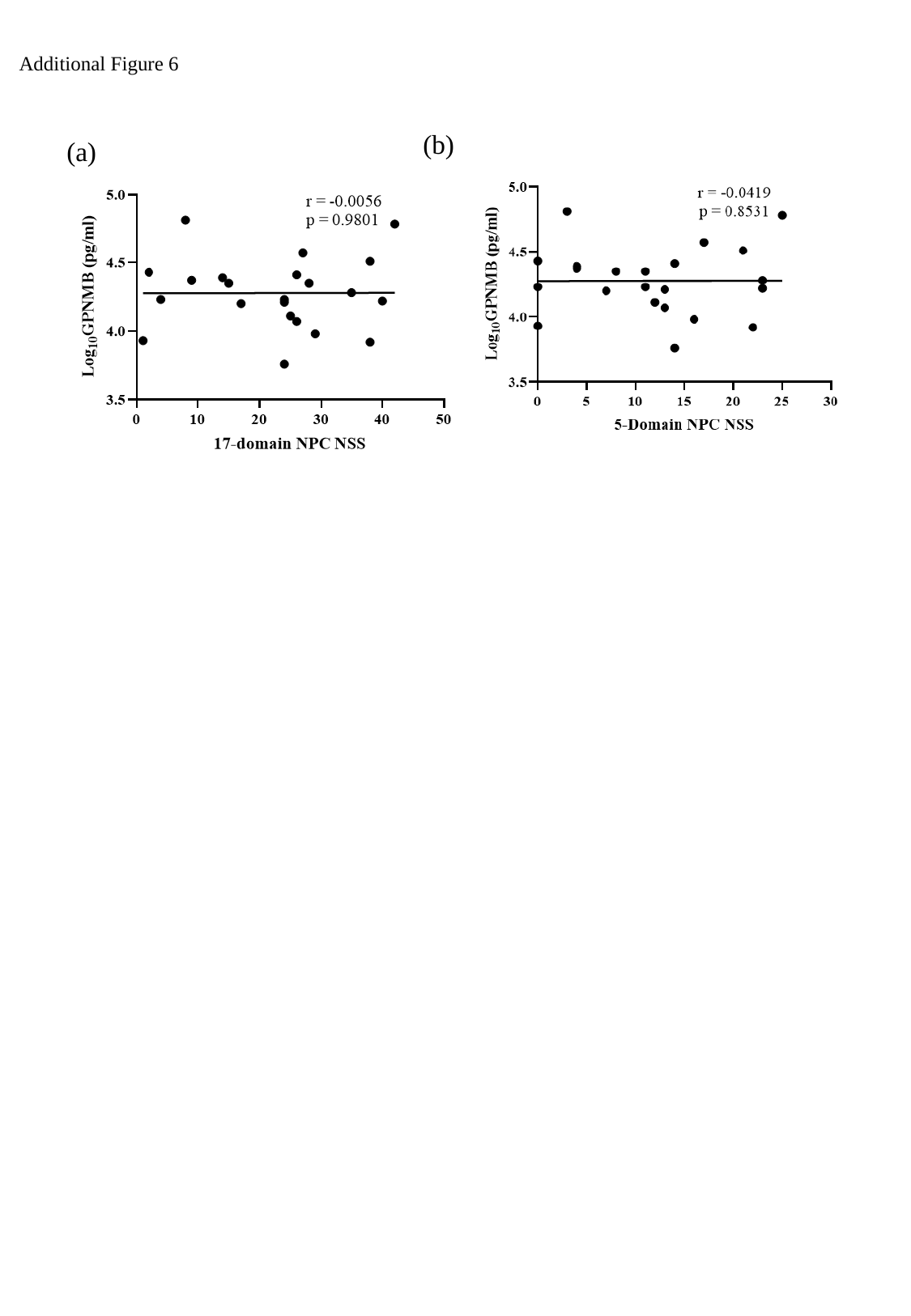

Additional Figure 6
(b)
(a)

## Slide 5
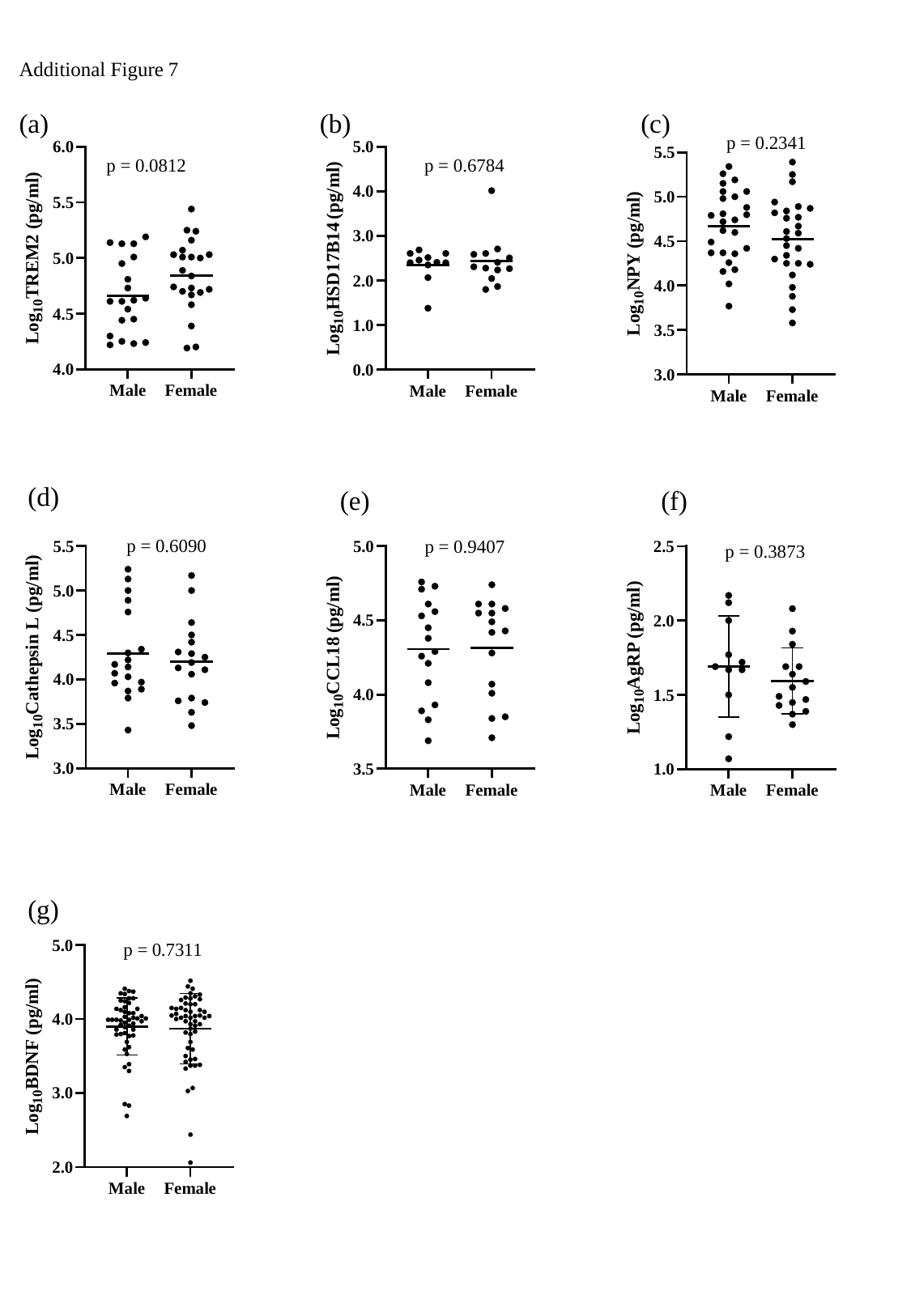

Additional Figure 7
(a)
(b)
(c)
(d)
(e)
(f)
(g)

## Slide 6
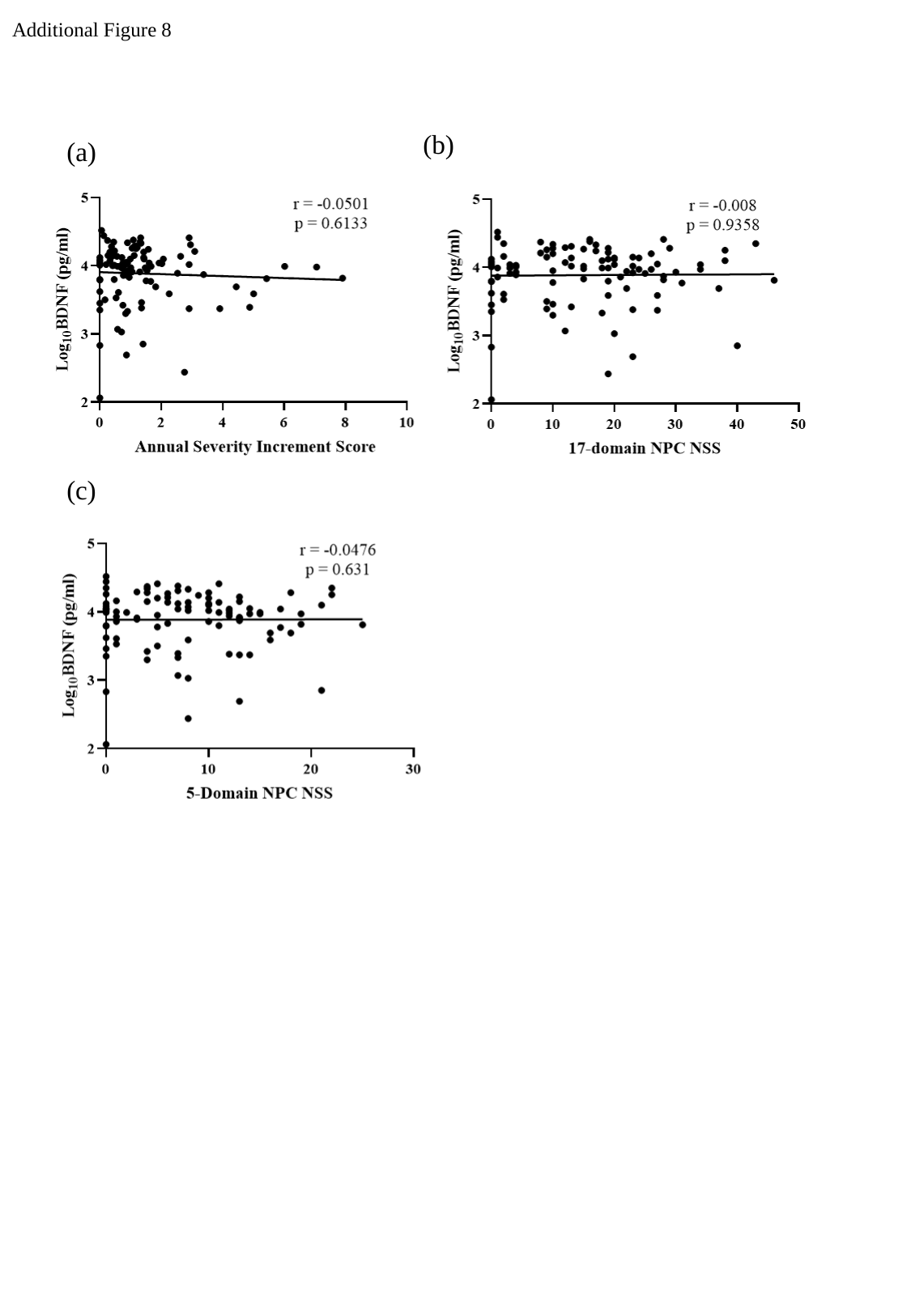

Additional Figure 8
(b)
(a)
(c)

## Slide 7
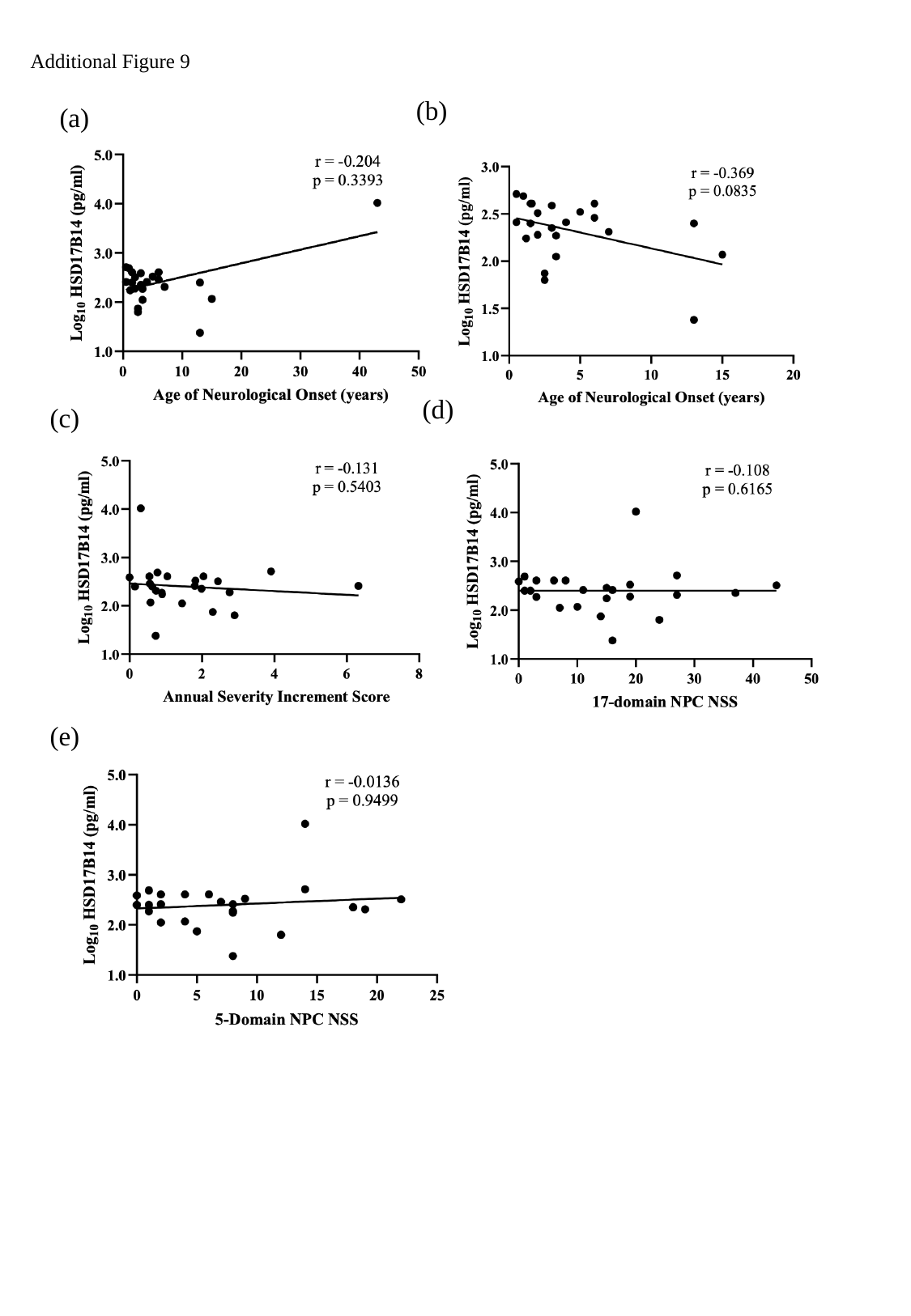

Additional Figure 9
(b)
(a)
(d)
(c)
(e)

## Slide 8
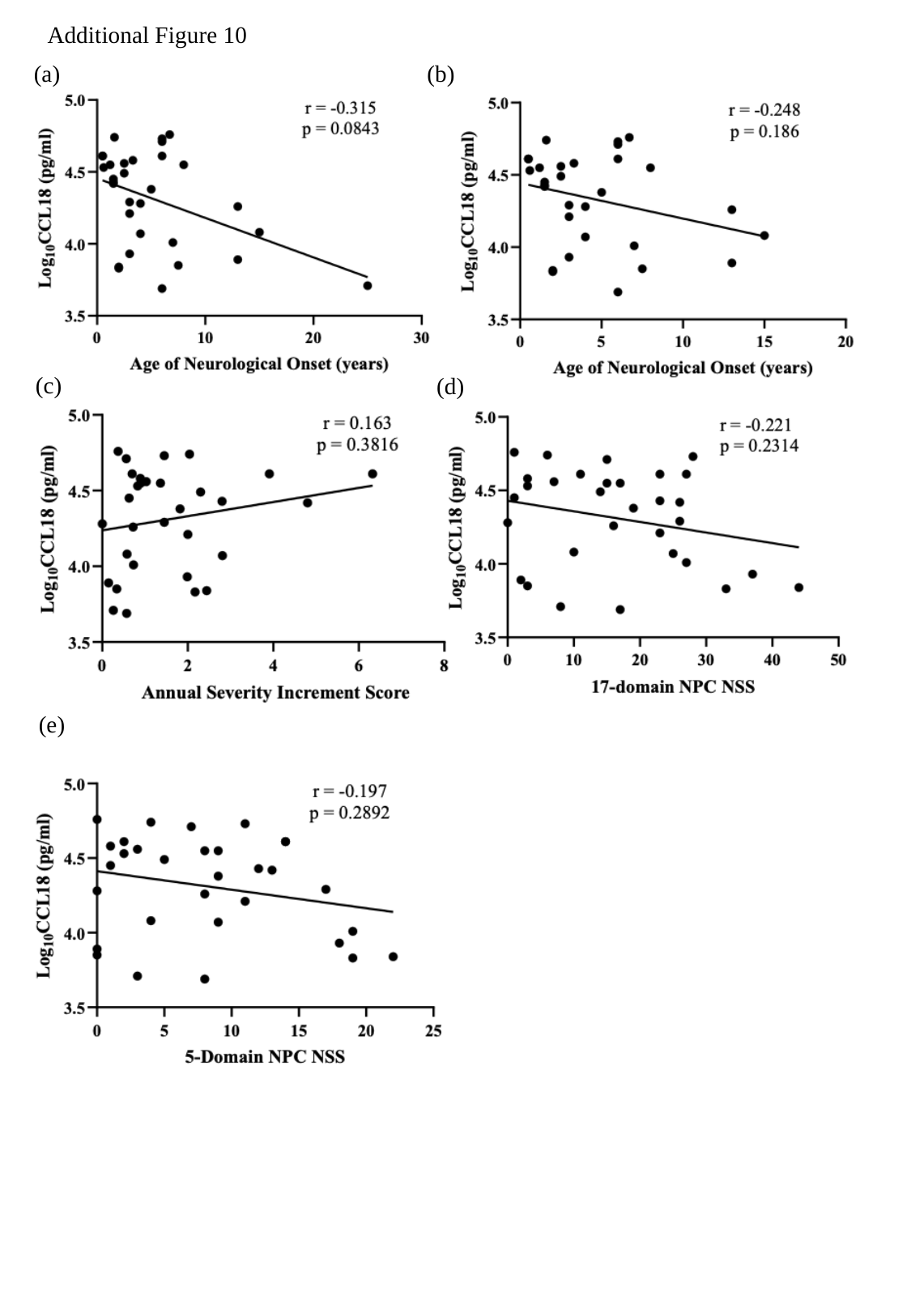

Additional Figure 10
(b)
(a)
(c)
(d)
(e)

## Slide 9
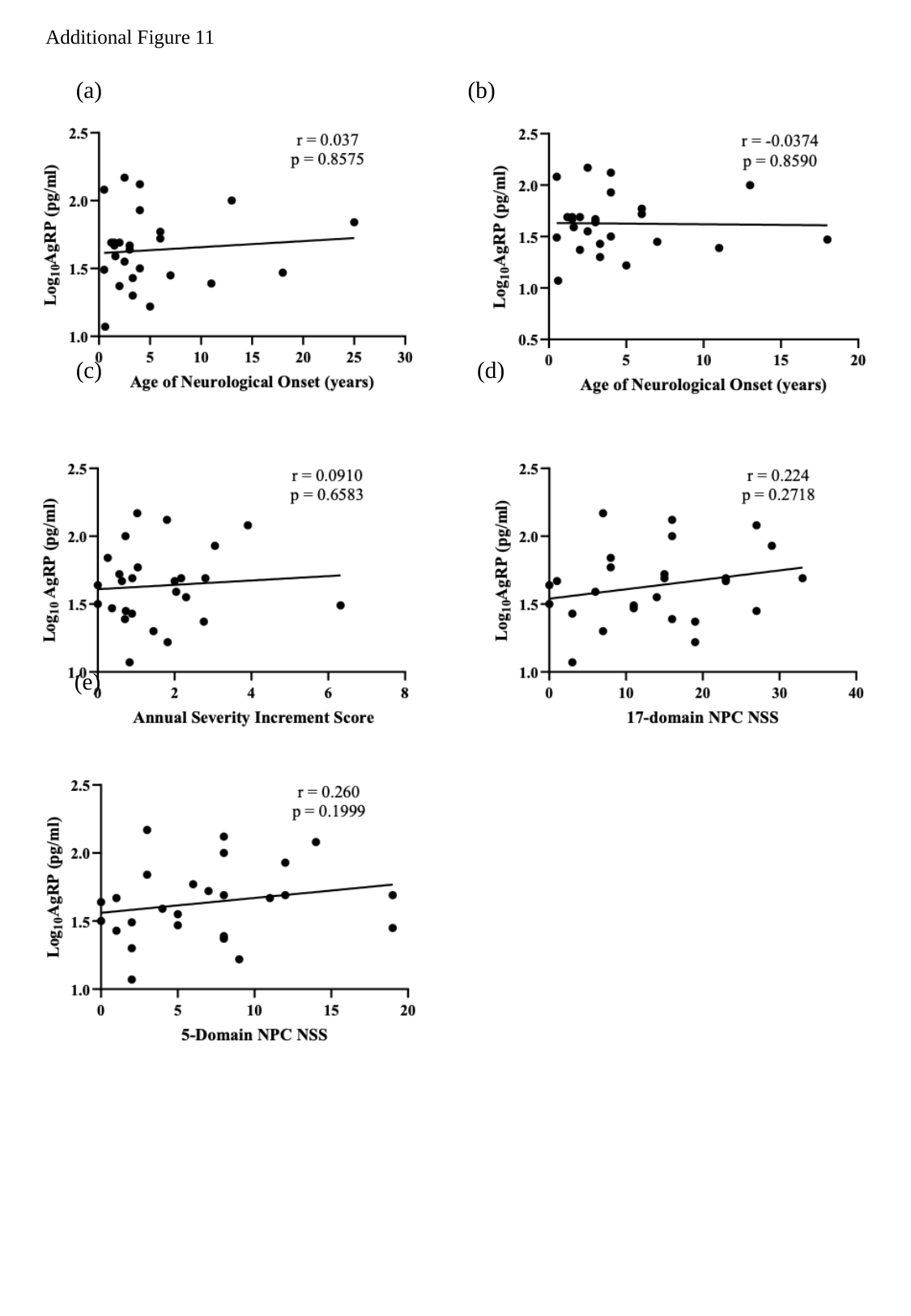

Additional Figure 11
(b)
(a)
(c)
(d)
(e)

## Slide 10
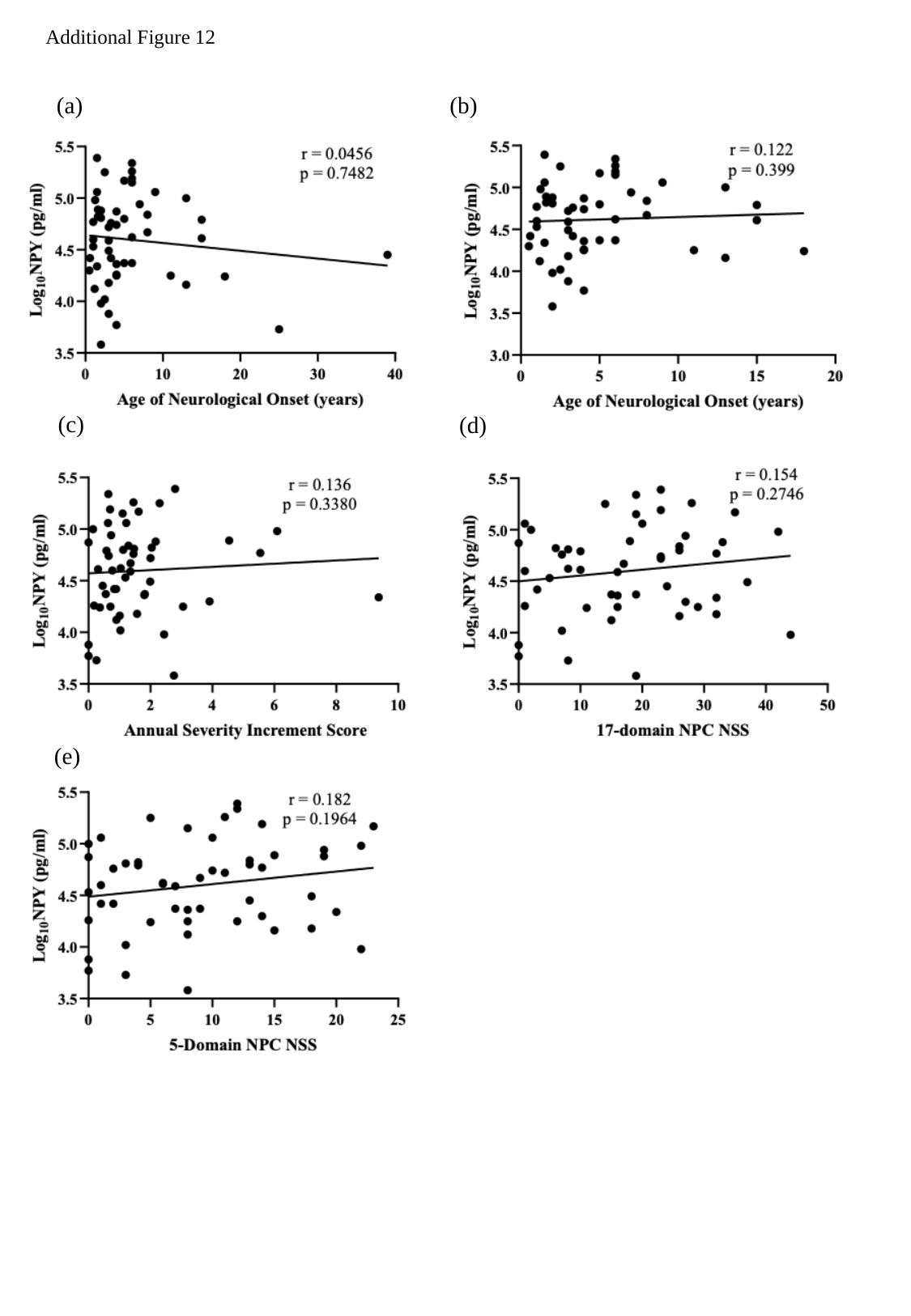

Additional Figure 12
(b)
(a)
(c)
(d)
(e)

## Slide 11
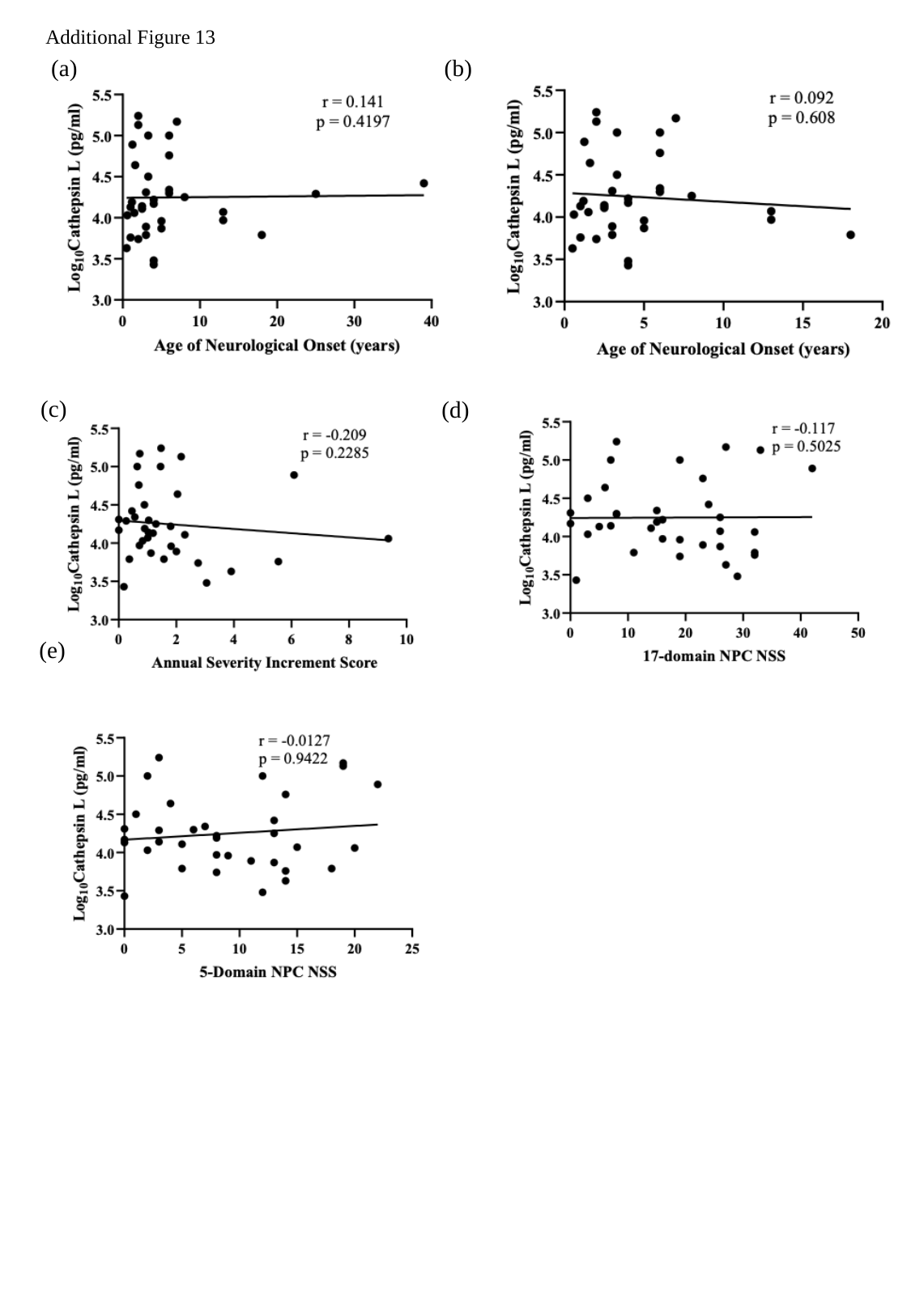

Additional Figure 13
(b)
(a)
(c)
(d)
(e)

## Slide 12
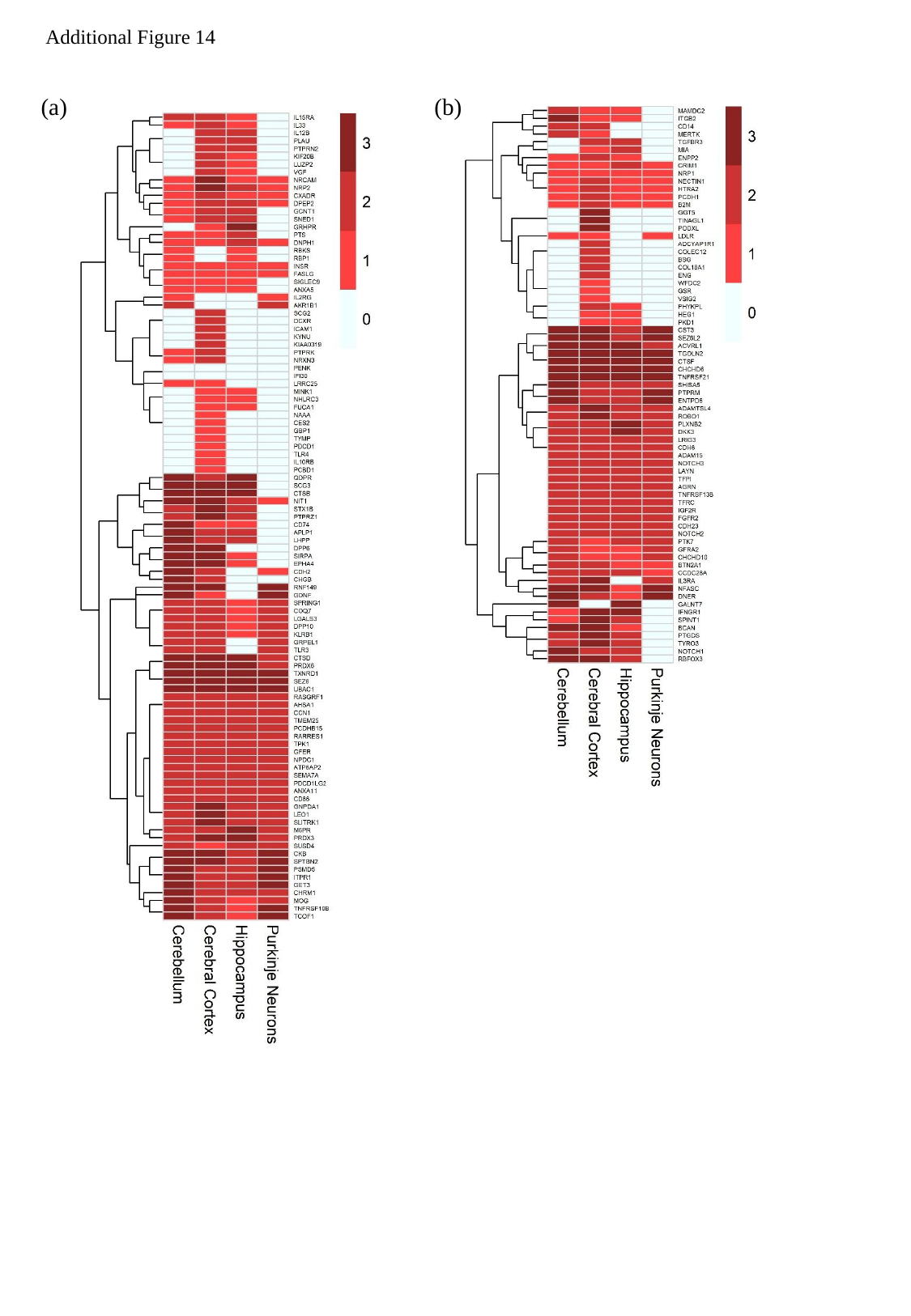

Additional Figure 14
(b)
(a)

## Slide 13
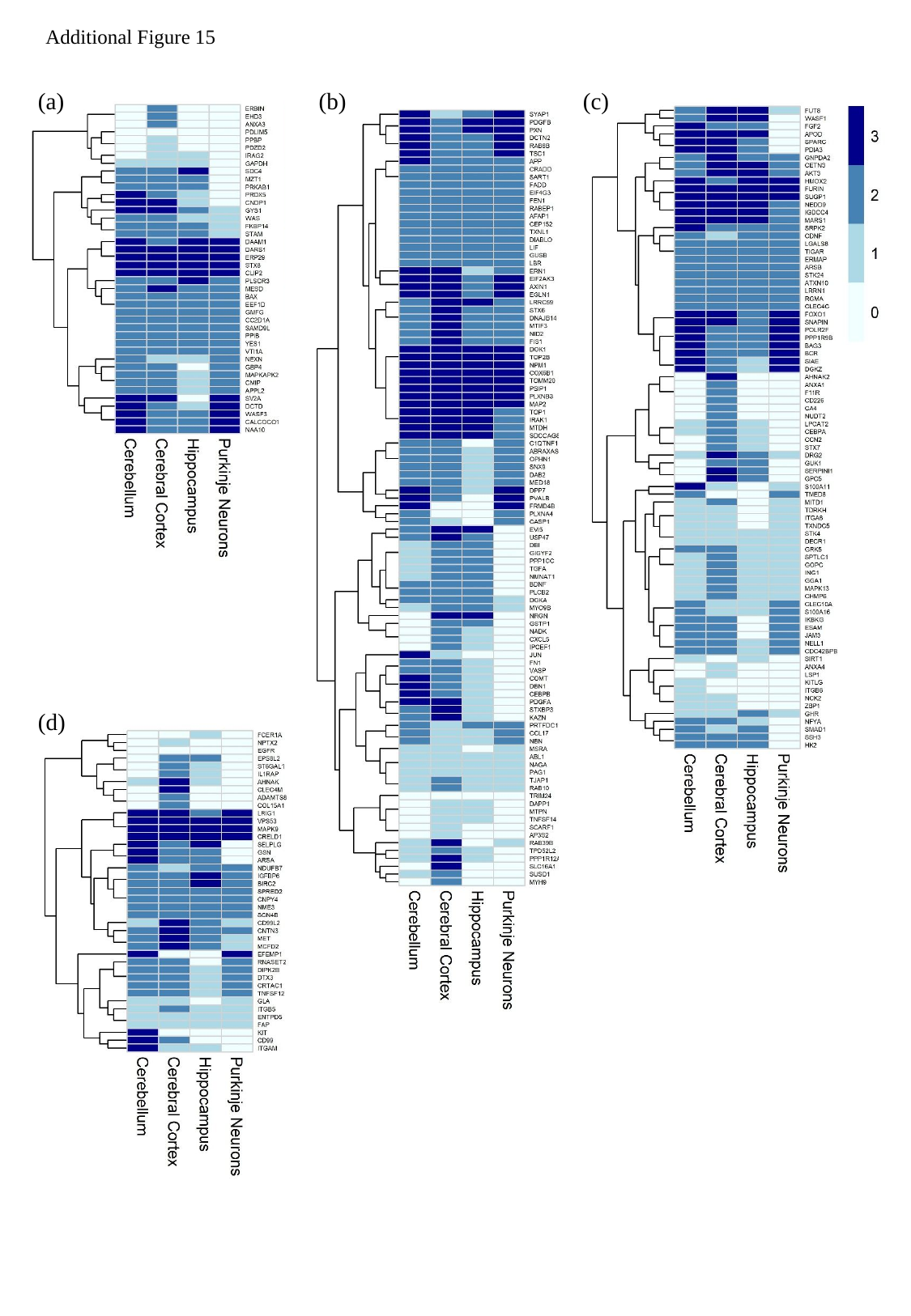

Additional Figure 15
(a)
(b)
(c)
(d)
